# Tailoring integrated care services for high-risk patients with multiple chronic conditions: a risk stratification approach using cluster analysis

**DOI:** 10.1186/s12913-020-05668-7

**Published:** 2020-08-27

**Authors:** Pablo E. Bretos-Azcona, Eduardo Sánchez-Iriso, Juan M. Cabasés Hita

**Affiliations:** 1 Universidad Pública de Navarra (UPNA), Campus de Arrosadia, s/n, 31006 Pamplona, Spain; 2 Instituto de Investigación Sanitaria de Navarra (IdiSNA), Calle Irunlarrea 3, 31008 Pamplona, Spain

**Keywords:** Risk stratification, Integrated care, Case management, Cluster analysis

## Abstract

**Background:**

The purpose of this study was to produce a risk stratification within a population of high-risk patients with multiple chronic conditions who are currently treated under a case management program and to explore the existence of different risk subgroups. Different care strategies were then suggested for healthcare reform according to the characteristics of each subgroup.

**Methods:**

All high-risk multimorbid patients from a case management program in the Navarra region of Spain were included in the study (*n* = 885). A 1-year mortality risk score was estimated for each patient by logistic regression. The population was then divided into subgroups according to the patients’ estimated risk scores. We used cluster analysis to produce the stratification with Ward’s linkage hierarchical algorithm. The characteristics of the resulting subgroups were analyzed, and post hoc pairwise tests were performed.

**Results:**

Three distinct risk strata were found, containing 45, 38 and 17% of patients. Age increased from cluster to cluster, and functional status, clinical severity, nursing needs and nutritional values deteriorated. Patients in cluster 1 had lower renal deterioration values, and patients in cluster 3 had higher rates of pressure skin ulcers, higher rates of cerebrovascular disease and dementia, and lower prevalence rates of chronic obstructive pulmonary disease.

**Conclusions:**

This study demonstrates the existence of distinct subgroups within a population of high-risk patients with multiple chronic conditions. Current case management integrated care programs use a uniform treatment strategy for patients who have diverse needs. Alternative treatment strategies should be considered to fit the needs of each patient subgroup.

## Background

Decades of progressive declines in the burden of communicable diseases and consequent improvements in life expectancy have shifted clinical and managerial concerns towards chronic illnesses, which are reaching alarming levels of prevalence in aging societies [1, 2].

Special attention has been given to multimorbidity [3, 4] and high-risk multiple chronic condition (MCC) patients in particular. Despite representing a small share of the chronic patient population, high-risk MCC patients account for a great share of healthcare organization budgets [5]. The elevated number of consultations, hospitalizations, and other treatments from different, uncoordinated specialties decreases favorable outcomes and increases cost [6].

Plans for appropriate management and delivery of care in the context of high-risk patients focus efforts on the realignment of systems towards case management integrated care programs [1, 7]. These models plan and coordinate care around specific high-risk patients through the assignment of a reference physician or a small multidisciplinary team. Teams assess the individual needs of each patient, develop a care plan accordingly and coordinate treatment delivery. Patients are monitored with periodic reassessments [8].

Identifying patients for which case management would be appropriate is an essential element of programs of this nature, and it is usually done by means of risk stratification techniques that classify patients with similar clinical needs into homogeneous groups [9]. This requires the establishment of a risk score using statistical models together with judgments from clinicians and the formation of certain thresholds for the assignment of patients to different risk strata [10].

In general terms, candidates for case management belong to the top 5% risk stratum of the population and are identified using a variety of ready-to-use risk stratification tools, including clinical risk groups (CRGs), adjusted clinical groups, diagnosis-related groups, diagnostic cost groups or the senior segmentation algorithm among others [9, 11–13]. A set of common services can be provided where risk stratification produces a homogeneous group of patients, and if needs are appropriately addressed, case management will fit patients in a cost-effective way, avoiding wasteful, unnecessary care.

However, evidence has shown that case management programs are not cost-effective in comparison to nonintegrated care programs for high-risk patients [7, 8, 14]. Case management interventions are not suitable for all high-risk patients but for a subset of patients who would benefit from them [14]. In other words, the population they target is heterogeneous and has different needs [10, 15–17], yet all patients are treated in a uniform manner under the same case management strategy. Since some groups of patients are receiving a type of care that does not fit their needs, care provides minimal or no health benefit to those patient subgroups and does not justify the costs, translating into low-value care for some of the patients in the high-risk population [18].

Therefore, case management requires further stratification to identify those patient subgroups that do not benefit from their current care and to adapt care strategies for them. The purpose is to target appropriate care for the appropriate patients. By reorganizing high-risk integrated care programs, we aim to target new services to selected groups of patients that are most likely to benefit from them. The extent to which newly organized services fit the clinical needs of patient subgroups will determine both improvements in outcomes and the degree of efficiency in healthcare resource utilization.

The purpose of this study was to produce a restratification within the high-risk MCC patient population, exploring the existence of different risk subgroups. Subsequently, the characteristics of each risk stratum were defined. Finally, we proposed different care strategies according to the risk profile of each subgroup, tailoring integrated care for high-risk patients.

## Methods

### Data and participants

In 2016, the Navarra region of Spain implemented an integrated care program for the treatment of chronic illness, which included a case management model for high-risk, noncancer MCC patients [19]. This study included all high-risk MCC patients who were treated in the region’s case management program from April 2016 – August 2018. The conditions to qualify for program enrollment were as follows:

Patients suffered at least three selected noncancer pathologies, including heart failure, dementia, ischemic heart disease, cerebrovascular disease, diabetes, chronic obstructive pulmonary disease, asthma, chronic renal failure and cirrhosis, and patients belonged to the top 5% of the risk pyramid according to the adjusted morbidity groups (GMA). GMA is a stratification tool similar to CRGs that is widely applied in Spain [20, 21]. A total of 885 patients were considered.

Data were obtained from the high-risk case management program database, which is anonymized and includes sociodemographic data, as well as data regarding functional status (Barthel score), nutritional status (serum albumin), renal deterioration status (creatinine, albumin/creatinine index), the presence of pressure skin ulcers, the number of prescriptions, prevalence and number of coexisting selected illnesses and the GMA risk score. In addition, the database also incorporates professionally rated variables such as clinical severity, nursing needs and social needs. A combination of the former is also available as global severity status. All variables were measured at patient inclusion in the program, when patients underwent a comprehensive assessment of their situation. Missing values were filled using multiple imputation to avoid biases in risk score estimation and subsequent stratification (Table 2) [22].

### Producing a risk score

A risk score was estimated for each of the patients using data from the initial comprehensive assessment that was completed upon inclusion in the case management program. The outcome for this risk score estimation was 1-year mortality from enrollment in the case management program. The reason why mortality was used as the outcome was that our population of interest consisted of patients with different chronic illness combinations. Therefore, disease-specific outcomes were not appropriate, as it was not possible to apply them to all patients under study. A common outcome was needed, and 1-year mortality was selected.

The risk score was estimated by logistic regression, where we first tested all variables in univariate analyses. Those variables that were significant were then fitted into a multivariate model, and insignificant variables were eliminated from the model in a stepwise manner. Significant predictors included the functional status, creatinine value, global severity status and presence of pressure skin ulcers. The results were validated using cross-validation techniques, as well as bootstrapping. A full description of the risk score estimation process is available in another published study [23]. Subsequently, patients were categorized into different ‘buckets’ or clusters according to their estimated risk [24].

### Risk stratification

For the purpose of determining patient subgroups and categorizing individuals into distinct, mutually exclusive risk strata, we used machine learning algorithms. These techniques group individuals who have similar risk scores into subgroups that are dissimilar and are more frequently termed cluster analysis [25].

**Fig. 1:**
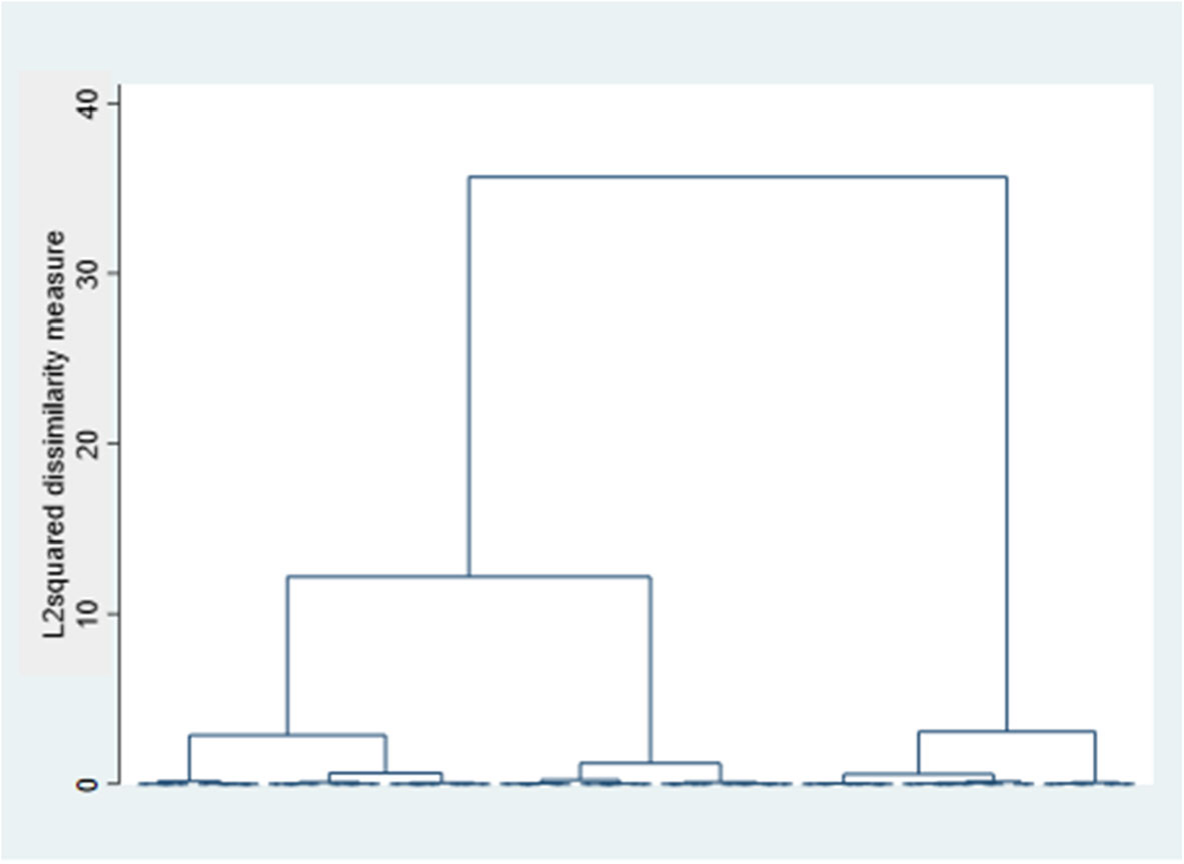
Patient grouping process (dendrogram)

We used Ward’s linkage hierarchical algorithm with the squared Euclidian distance (L2squared). The optimal number of clusters was determined using the Duda/Hart stopping rule and visually through a dendrogram (Fig. 1). A dendrogram is a diagram that shows how observations merge with other observations that are similar to them in terms of distance. Those observations that are closest to them, or equivalently those that have the most similar risk scores, are merged into a group. This process continues iteratively, and larger, distinct groups can be observed in the dendrogram. Mergers are represented as nodes, and the distance between groups of patients is shown in the vertical axis. The results were validated using silhouettes, reassigning individuals to a different cluster when needed [26].

To evaluate the stability of the results, the full sample was randomly divided into four equally sized subsamples, each containing 25% of the observations, and the algorithm was run again on each subsample. It is possible to think about this process as k-fold cross validation with four folds. The robustness of the results was further tested by running the K-means algorithm, setting the parameter k equal to Ward’s linkage optimal number of clusters. Table 1 shows how many patients belong to each cluster when using the two clustering techniques used in this study, Ward’s linkage algorithm, and K-means algorithm that was performed as a robustness check. In addition, the number of patients in each of the four randomly divided subsamples from stability analysis is shown.

**Table 1:** Distribution of patients among clusters

	Cluster 1	Cluster 2	Cluster 3
Ward’s linkage (n = 885)	400 (45.20%)	336 (37.96%)	149 (16.84%)
Stability Analysis			
Sub-Sample 1 (*n* = 221; 25%)	91(41.18%)	91(41.18%)	39(17.64%)
Sub-Sample 2 (n = 221; 25%)	98 (44.34%)	89 (40.28%)	34 (15.38%)
Sub-Sample 3 (n = 221; 25%)	105 (47.52%)	74 (33.48%)	42 (19.00%)
Sub-Sample 4 (*n* = 222; 25%)	100 (45.05%)	88 (39.64%)	34 (15.32%)
Total (n = 885)	394 (44.52%)	342 (38.64%)	149 (16.84%)
K-means (n = 885)	370 (41.81%)	348 (39.32%)	167 (18.87%)

### Cluster examination

Following the identification of patient subgroups, their clinical and sociodemographic characteristics were compared to test if there were significant differences between them. The prevalence of chronic illness diagnoses and their most frequent combinations were also compared.

When considering continuous variables, one-way ANOVA tests were performed. Kruskal-Wallis tests were used when considering categorical variables or if the assumptions for ANOVA did not hold, and a χ2 test was used for binary variables. If significant differences in patient characteristics were observed across clusters, further post hoc pairwise tests were completed to detect which cluster was different from the remaining clusters. Multiple one-way ANOVA comparisons with Bonferroni corrections, Mann-Whitney U tests and Fisher’s exact tests were used if ANOVA, Kruskal-Wallis or χ2 tests were used, respectively. All analyses were carried out using STATA 15.0 software.

## Results

### Clustering results

Both the Duda/Hart stopping rule and the clustering process dendrogram (Fig. 1), which shows the last 100 grouping nodes, indicated the presence of three distinct clusters within the high-risk MCC patient population. The optimal number of clusters was also assessed considering its clinical relevance and interpretability.

The clusters were ordered in such a way that the 1-year mortality risk scores were incremental. Hence, patients with the lowest risk scores belong to cluster 1, cluster 2 includes intermediate cases, and very high-risk patients have been allocated to cluster 3. With respect to the distribution of patients among clusters, cluster 1 included ≃45% of patients, ≃38% were classified into cluster 2, and ≃17% were assigned to cluster 3.

The structure of the data remained constant in the stability analysis, showing that the patterns in the subgroup distribution were reproducible even if random parts of the sample were excluded. Table 1 shows one of the many random partitions that were carried out, all with very similar results. The K-means algorithm also showed a similar pattern in the data, producing roughly the same patient distribution among clusters. Generally, these robustness checks confirmed that the 3-cluster solution and the resulting proportion of patients assigned to each cluster were robust.

### Cluster characteristics

The representative features of each cluster are described in Tables 2 and 3. We report mean values or proportions, together with the significance test *p*-values and post hoc test results.

**Table 2:** Patient Characteristics within Clusters

	Total population n = 885	Cluster 1 ***n*** = 400	Cluster 2 ***n*** = 336	Cluster 3 ***n*** = 149	ANOVA/Kruskal-Wallis/χ2 test
**1-year mortality risk score (%)**	32.15 ± 18.04%	16.95 ± 6.45%	36.64 ± 6.67%	62.84 ± 10.68%	KW < 0.001^a^
**Age (mean ± s.d.)**	83.33 ± 8.37	81.16 ± 8.51	84.32 ± 8.05	86.89 ± 6.97	KW < 0.001^a^
**Sex (% Males)**	56%	62%	55%	44%	χ2 < 0.001^c^
**Informal Caregiver (%)**	91%	88%	94%	93%	χ2: 0.011^d^
**GMA score (mean ± s.d.)**	22.81 ± 6.82	22.47 ± 6.39	23.04 ± 7.06	23.24 ± 7.33	AN: 0.379
**Barthel scale (mean ± s.d.)**	59.95 ± 29.45	82.03 ± 18.16	51.64 ± 17.24	19.40 ± 22.15	KW < 0.001^a^
**Albumin/creatinine index (mean ± s.d.)**	1.49 ± 0.67	1.49 ± 0.66	1.51 ± 0.70	1.46 ± 0.66	KW: 0.792
**≤30 mg/g: Normal (n, %)**	540 (61%)	242 (61%)	203 (60.42%)	95 (64%)	
**30–300 mg/g: Moderate (n, %)**	254 (29%)	120 (30%)	94 (28%)	40 (27%)	
**≥300 mg/g: High (n, %)**	91 (10%)	38 (9%)	39 (12%)	14 (9%)	
**Creatinine (mg/dL) (mean ± s.d.)**	1.52 ± 0.78	1.40 ± 0.59	1.57 ± 0.75	1.72 ± 1.16	KW: 0.002^b^
**Serum albumin (g/dL) (mean ± s.d.)**	3.80 ± 0.43	3.90 ± 0.37	3.76 ± 0.41	3.59 ± 0.50	KW < 0.001^a^
**Pressure skin ulcers (%)**	28%	21%	26%	55%	χ2 < 0.001^c^
**Number of prescriptions (mean ± s.d.)**	8.08 ± 3.55	7.82 ± 3.48	8.47 ± 3.75	7.95 ± 3.20	AN: 0.038^d^
**Intake of opioids (%)**	15%	17%	12%	13%	χ2: 0.168
**Intake of psycholectics (%)**	8%	5%	9%	16%	χ2 < 0.001^c^
**Global severity (mean ± s.d.)**	2.87 ± 0.45	2.63 ± 0.50	2.99 ± 0.09	3.22 ± 0.41	KW < 0.001^a^
**Mild (n, %)**	4 (< 1%)	4 (1%)	–	–	
**Moderate (n, %)**	144 (16%)	141 (35%)	3 (1%)	–	
**Severe (n, %)**	704 (80%)	25 (64%)	333 (99%)	116 (78%)	
**Very severe (n, %)**	33 (4%)			33 (22%)	
**Clinical severity (mean ± s.d.)**	3.30 ± 0.68	3.09 ± 0.75	3.43 ± 0.59	3.58 ± 0.55	KW < 0.001^a^
**Mild (n, %)**	2 (< 1%)	2 (1%)	–	–	
**Moderate (n, %)**	110 (12%)	89 (22%)	17 (5%)	4 (3%)	
**Severe (n, %)**	393 (44%)	180 (45%)	158 (47%)	55 (37%)	
**Very severe (n, %)**	380 (43%)	129 (32%)	161 (48%)	90 (60%)	
**Nursing needs (mean ± s.d.)**	2.65 ± 0.88	2.23 ± 0.80	2.82 ± 0.77	3.41 ± 0.66	KW < 0.001^a^
**Mild (n, %)**	76 (9%)	66 (16%)	10 (3%)	–	
**Moderate (n, %)**	323 (36%)	204 (51%)	105 (31%)	14 (10%)	
**Severe (n, %)**	318 (36%)	103 (26%)	155 (46%)	60 (40%)	
**Very severe (n, %)**	168 (19%)	27 (7%)	66 (20%)	75 (50%)	
**Social needs (mean ± s.d.)**	1.77 ± 0.70	1.71 ± 0.68	1.82 ± 0.71	1.84 ± 0.73	KW: 0.095
**Mild (n, %)**	327 (37%)	162 (40%)	116 (35%)	49 (33%)	
**Moderate (n, %)**	451 (51%)	199 (50%)	173 (51%)	79 (53%)	
**Severe (n, %)**	90 (10%)	33 (8%)	40 (12%)	17 (11%)	
**Very severe (n, %)**	17 (2%)	6 (2%)	7 (2%)	4 (3%)	

*KW* Kruskal-Wallis test, *AN* ANOVA, *χ2* Chi-squared test

Missing values that were imputed for analysis (% missing): creatinine (0.23%), albumin/creatinine index (2.15%) clinical severity (3.62%), nursing needs (9.38%), global severity (10.96%), Barthel scale (14.24%), serum albumin (15.71%), social needs (43.62%)

^a^All clusters are significantly different from each other

^b^Cluster 1 is significantly different to the remaining clusters

^c^Cluster 3 is significantly different to the remaining clusters

^d^Clusters 1 and 2 are significantly different, but there were no other differences in the remaining pairwise post hoc tests

**Table 3:** Patient Diagnoses within Clusters

	Total population ***n*** = 885	Cluster 1 ***n*** = 400	Cluster 2 ***n*** = 336	Cluster 3 ***n*** = 149	
**Number of comorbidities (mean ± s.d.)**	3.65 ± 0.81	3.66 ± 0.82	3.67 ± 0.84	3.61 ± 0.75	KW: 0.928
**Prevalence of illnesses**					**χ2 test**
Diabetes (n, %)	635 (72%)	289 (72%)	237 (71%)	109 (73%)	p: 0.803
Chronic Renal Failure (n, %)	591 (67%)	257 (64%)	234 (70%)	100 (67%)	p: 0.301
Ischemic Heart Disease (n, %)	444 (50%)	212 (53%)	169 (50%)	63 (42%)	p: 0.082
Heart Failure (n, %)	595 (67%)	267 (67%)	234 (70%)	94 (63%)	p: 0.352
Cerebrovascular Disease (n, %)	283 (32%)	113 (28%)	106 (32%)	64 (43%)	p: 0.004^a^
COPD (n, %)	263 (30%)	135 (34%)	102 (30%)	26 (17%)	p: 0.001^a^
Asthma (n, %)	190 (21%)	98 (25%)	66 (20%)	26 (17%)	p: 0.118
Dementia (n, %)	166 (19%)	56 (14%)	60 (18%)	50 (34%)	*p* < 0.001^a^
Cirrhosis (n, %)	68 (8%)	36 (9%)	26 (8%)	6 (4%)	p:0.151
**Most frequent illness combinations**					
Diabetes + Chronic Renal Failure + Heart Failure (n, %)	67 (8%)	26 (7%)	29 (9%)	12 (8%)	p: 0.537
Diabetes + Chronic Renal Failure + Heart Failure + Ischemic Heart Disease (n, %)	50 (6%)	20 (5%)	19 (6%)	11 (7%)	p: 0.561
Chronic Renal Failure + Heart Failure + Ischemic Heart Disease (n, %)	36 (4%)	16 (4%)	16 (5%)	4 (3%)	p: 0.563
Diabetes + Chronic Renal Failure + Ischemic Heart Disease (n, %)	34 (4%)	16 (4%)	16 (5%)	2 (1%)	p: 0.190
Diabetes + Heart Failure + Ischemic Heart Disease (n, %)	25 (3%)	14 (4%)	5 (1%)	6 (4%)	p: 0.162
Diabetes + Heart Failure + COPD (n, %)	25 (3%)	13 (3%)	8 (2%)	4 (3%)	p: 0.773
Chronic Renal Failure + Heart Failure + COPD	17 (2%)	10 (3%)	6 (2%)	1 (1%)	p: 0.372
Heart Failure + Ischemic Heart Disease + Cerebrovascular Disease (n, %)	18 (2%)	5 (1%)	10 (3%)	3 (2%)	p: 0.255
Diabetes + Heart Failure + Cerebrovascular Disease (n, %)	18 (2%)	7 (2%)	7 (2%)	4 (3%)	p: 0.786
Diabetes + Chronic Renal Failure + Heart Failure + Asthma (n, %)	15 (2%)	10 (3%)	2 (1%)	3 (2%)	p: 0.130

*χ2* Chi squared test

^a^Cluster 3 is significantly different to the remaining clusters

Some of the reported variables varied across clusters. Age increased significantly from one cluster to the next. The Barthel scale was significantly different for all patient types, showing extensive declines in functional status from cluster to cluster. Moreover, serum albumin values were also significantly different across clusters, indicating poorer nutritional status. The majority of professional-rated variables increased significantly among clusters, as shown by global status, clinical severity and nursing needs. Social needs were the exception in this group of variables, as no significant differences were reported.

We can therefore say that mortality risk scores increase as age progresses, alongside a deterioration of functional status, nutritional values, clinical severity status and nursing needs status (Table 2). While these trends are common to all patients, certain features inherent to particular clusters were observed:

- **▪ Cluster 1**: risk scores [0–26.70%]
  Patients in this cluster showed a lower renal deterioration degree, as measured by creatinine, in comparison to the rest of the clusters. The number of prescriptions and the proportion of patients who had an informal caregiver were significantly lower than those of cluster 2, but there were no significant differences with respect to cluster 3.
- **▪ Cluster 2**: risk scores [26.70–50.80%]
  No particular differences were reported with respect to the other clusters, apart from the common differences that relate to age, functional status, nutritional values and the professional-rated variables mentioned above.
- **▪ Cluster 3**: risk scores [50.80–100%]
  All patients included in this subgroup had a higher likelihood of dying than of surviving the following year. Regarding specific cluster features, we found a higher proportion of female patients than in other subgroups. Patients included in this subgroup presented a notable increase in the existence of pressure ulcers, with more than 50% of them presenting this problem. Regarding diagnosis, patients in this cluster presented a significantly lower prevalence rate of COPD but higher prevalence rates of cerebrovascular disease and dementia in comparison to those in clusters 1 and 2. In line with the higher prevalence of dementia, a higher intake of psycholectics also was observed.

Despite the many differences described above, some other characteristics remained unchanged across clusters. This was the case for the albumin/creatinine index, which is an early screener for kidney disease, the intake of opioids, and social needs, as highlighted earlier.

The GMA risk score, which was the metric used to select the top 5% of risks for our study, was similar for all clusters. That is, while the mortality risk scores varied among different clusters, the GMA risk scores remained the same.

All other diagnoses apart from COPD, cerebrovascular disease and dementia had similar prevalence rates among the clusters. Regarding the most frequent illness combinations present in our population, no significant differences across clusters were observed (Table 3). The number of coexisting chronic conditions was also equal in all subgroups.

## Discussion

This study demonstrates the existence of clinically distinct subgroups within the a population of high-risk patients with multiple chronic conditions, confirming that case management integrated care programs use a uniform treatment strategy for patients who have diverse needs. That is, case management treats heterogeneous populations in a homogeneous way.

The need for a data-based, high-risk patient stratification has been extensively illustrated in the literature, but despite its potential it remains underdeveloped, and only a few studies exist [27]. One of the reasons why this may be the case is that proprietary stratification algorithms such as CRGs are already in place, so healthcare professionals or managers do not see the necessity of using alternative approaches. However, while these algorithms provide considerably better solutions than demographic approaches, they are poor risk adjusters when mortality and other clinical outcomes are considered [21, 24]. There is a lack of alignment between the purpose of proprietary algorithms, which aim to stratify patient populations based on estimates of future healthcare resource consumption [13], and the purpose of this study, that is, to stratify patients according to their clinical needs.

Our results support these statements and show how the GMA score, the Spanish equivalent of CRGs, does not vary across subgroups, whereas mortality risk scores do differ from cluster to cluster. GMA does not offer the desired level of granularity to observe clinically relevant subpopulations among high-risk patients, resulting in a homogeneous population from a cost point of view, while subpopulations with different needs remain undetected if alternative risk stratification methods are not introduced.

Given that ready-to-use risk stratification tools are not adequate for the purposes of this study, alternative segmenting methods were explored. Big data techniques and cluster analysis in particular have been proposed for these purposes in the literature when electronic records are available, as in our case [9, 16, 28]. We showed that cluster analysis is a useful tool for producing risk stratifications, providing valuable information for healthcare reform and robust results that are easy to interpret.

With respect to the variables that were used to stratify our population, only clinically related and demographic variables were used. This approach offers several advantages, emphasizing relevant health priorities that should be addressed and informing the design of new services or the reform of the existing ones [27]. In contrast, the demand for healthcare services does not always inform areas of clinical concern but of cost concern. Health reforms that arise from using utilization rates for risk stratification may go against the interest of the patient, since the aim of the policy maker may be to reduce costs instead of improve population health [29].

Moreover, if utilization rates are to be used, episodes of care should be comparable [24]. All patients should suffer the same health problem or diagnosis, and all demand episodes should be related to the medical area of interest and equally intense or of the same nature. If the former conditions are fulfilled, the quantity of care provided is appropriate for risk stratification. However, this is hardly ever the case, especially in the case of chronic illnesses, and in our study in particular, patients suffered different illness combinations or types of exacerbations, making utilization episodes incomparable.

A limitation of our study is that the population under study suffered from a specific set of chronic illnesses that may not be the same in other settings. In addition, patients only qualified for enrollment if they suffered from three or more chronic illnesses. Other programs may require only two chronic illnesses for enrollment. This may impact the generalizability of our study results. Moreover, we tested the cluster stability and robustness internally rather than externally. As a final limitation, we specified patient clusters using our own risk score estimations. However, different risk scoring models are likely to be used in other environments. We encourage others to reproduce our analyses and estimate risk scores for each context.

One study by Vuik et Al. [30] stratified a high-risk patient population using cluster analysis. However, that study grouped patients according to their utilization patterns and not their clinical risk scores or needs. Moreover, cancer patients were included while we did not include this type of patient. Four main subgroups among which care usage had significant variation were identified. Low et Al. [31] also provided a risk stratification using cluster analysis, using utilization data to group patients. Their study was not restricted to high-risk patients and included all types of adult patients in the analysis, without making distinctions in terms of their risk category or clinical profile. Five clusters were found.

Other studies that segment patient populations are available in the literature, although they used expert criteria to produce the resulting subgroups instead of data-driven approaches [17, 32]. Lynn et al. describe three end-of-life subgroups for frail, high-risk patients, which is in line with our results.

### Tailoring integrated care services

To date, all patients included in this study have been treated under the same case management strategy. Nevertheless, three distinct subgroups with different characteristics were identified for which care programs should be tailored.

We proposed a different care strategy for each type of patient so that treatment can adequately meet patient needs. These strategies were based on a literature review and supported by expert consultation with healthcare authorities from the region who have extensive experience with the integrated care program under study.

Patients included in cluster 1, whose risk status was the lowest of all subgroups and who had moderate functional status, severity status and nursing needs, could benefit from their current case management program. This program includes a reference specialist team that keeps patient follow-up, self-care education and support, a link nurse that is available 24 h by phone, and most importantly, direct hospital admission without passing through emergency services and a day hospital unit. At-home services are also available in some cases. All professionals develop personalized care plans that focus on avoiding exacerbations and sustaining function.

As health starts to decline in combination with a worsening functional status and increasing nursing needs, patients become increasingly dependent on a 3rd person, and transitions from home to the hospital can be complicated. Patients in cluster 2 would benefit from home-based programs that focus on improving quality of life and averting unnecessary hospitalizations or readmissions [32]. Mobile integrated care programs should be implemented for these purposes [33]. Nursing services, together with caregiver training and support, play an important role.

Those included in cluster 3, with the highest mortality risk scores, are very likely to die in the near future. Continuing functional declines, together with worsening clinical severity and other characteristics such as increases in pressure skin ulcers or higher rates of dementia and cerebrovascular disease, are indicators of the short survival prospect of patients included in this subgroup. Healthcare services should be directed towards end-of-life care, including hospices, or home-based palliative care services that shift attention from curative efforts to quality of life improvements [34].

A risk score estimation tool has been created for use in clinical practice to estimate patient risk scores [23]. This tool consists of a nomogram, which is a graphical calculation tool that synthesizes logit model results in a graph that is filled in by healthcare professionals and provides individual risk scores for each patient without the need for computers or software. Risk scores, in combination with the results of this study, can be used in clinical practice for patient classification purposes. Risk score calculation and subsequent patient classification should be performed at patient enrollment in the program but also at regular intervals or if healthcare professionals see it as necessary. This would allow close monitoring of each patient situation, providing valuable information that can assist treatment strategy decisions.

This study is a key part of the design of alternatives to case management care programs. By stratifying the population into differentiated subpopulations, we identified relevant patient types and their needs. The description of the characteristics of each patient type can guide the development of these new services. Moreover, study results provide valuable information for healthcare professionals in relation to the development of each patient’s condition and can assist treatment strategy decisions.

The extent to which patient outcomes such as mortality rates or quality of life improve will be determined in future research when alternative programs are implemented and their performance measured. The efficiency of new care strategies also needs to be measured in future research through cost-effectiveness analyses.

## Conclusions

This study produced a restratification for a population of high-risk multimorbid patients who are currently included in a case management integrated care program. We showed that the high-risk population had heterogeneous needs but that all patients received the same treatment. Risk stratification was performed using cluster analysis. The characteristics of each cluster were presented, outlining the specific needs that should be addressed in healthcare reform. We suggested alternatives to case management services that can make meaningful contributions to health outcomes, moving away from low-value care.

## Data Availability

The data that support the findings of this study are available from Servicio Navarro de Salud but restrictions apply to the availability of these data, which were used under license for the current study, and so are not publicly available. Data are however available from the authors upon reasonable request and with permission of Servicio Navarro de Salud.

## Abbreviations

MCC: Multiple Chronic Condition
CRGs: Clinical Risk Groups
GMA: Adjusted Morbidity Groups / Grupos de Morbilidad Ajustados in Spanish
COPD: Chronic Obstructive Pulmonary Disease
KW: Kruskal-Wallis
AN: ANOVA
